# A transfer learning-based multimodal model for early prediction of 90-day respiratory failure in dermatomyositis-associated interstitial lung disease

**DOI:** 10.3389/fimmu.2026.1867606

**Published:** 2026-07-16

**Authors:** Lihui Guo, Yaning Yao, Qirui Wu, Hui Wang, Caiyun Niu, Gang Wang, Fei Chen

**Affiliations:** 1 Department of Ultrasound, The First Hospital of Lanzhou University, Lanzhou, Gansu, China; 2 Information Center, The First Hospital of Lanzhou University, Lanzhou, Gansu, China; 3 Department of Radiology, The First Hospital of Lanzhou University, Lanzhou, Gansu, China

**Keywords:** artificial intelligence, dermatomyositis, interstitial lung disease, machine learning, multimodal prediction model, respiratory failure, transfer learning

## Abstract

**Background:**

Dermatomyositis-associated interstitial lung disease (DM-ILD) is a life-threatening condition that often leads to respiratory failure, poor prognosis, and high mortality, especially in patients positive for anti-melanoma differentiation-associated gene 5 (anti-MDA5) antibodies. Early identification of MDA5 positivity is crucial for timely intervention, yet routine testing is not consistently available across different regions. In some centers, the test is omitted or sent to external laboratories, which can delay recognition of disease severity and postpone appropriate treatment. Because risk assessment based on admission data remains limited, we focused on 90-day respiratory failure as an early and clinically relevant outcome, collecting patients’ clinical information within the first 48 hours of admission while excluding anti-MDA5 results. Using these data, we developed and internally tested a multimodal model to predict 90-day respiratory failure in patients with DM-ILD.

**Methods:**

In this exploratory single-center retrospective study, 124 adult patients with DM-ILD were analyzed using a 7:3 train-test split, with five-fold cross-validation used within the training set. Baseline predictors included demographic characteristics, clinical features, laboratory results, pulmonary function indices, and latent computed tomography (CT) features extracted from chest CT scans using a pre-trained model, all collected within 48 hours of admission. Multiple dimensionality-reduction, modeling, and fusion strategies were compared to identify the optimal framework. We evaluated model performance using the area under the receiver operating characteristic curve (AUC) and assessed interpretability with SHapley Additive exPlanations (SHAP).

**Results:**

Among the prespecified candidate models, the early-fusion random forest model incorporating principal component analysis (PCA) showed the best discriminative performance in the held-out test set, with an AUC of 0.967 and a PR-AUC of 0.879. SHAP analysis showed that arthritis, pulmonary function indices, laboratory markers, and several latent CT features were among the most influential predictors.

**Conclusions:**

The admission-based multimodal model demonstrated encouraging performance in internal testing and may aid early stratification of 90-day respiratory failure risk in patients with DM-ILD, particularly when anti-MDA5 antibody results are unavailable or delayed.

## 1 Introduction

Patients with dermatomyositis-associated interstitial lung disease (DM-ILD) often exhibit rapid disease progression and are prone to acute, severe respiratory failure (1, 2). In particular, anti-melanoma differentiation-associated gene 5 (anti-MDA5) antibody-positive DM is a severe subtype with an aggressive clinical course and high mortality. Anti-MDA5 antibody positivity is strongly associated with rapidly progressive interstitial lung disease (RP- ILD) (3, 4). Reported mortality rates of RP-ILD range from 33% to 59% (5, 6). Early recognition of high-risk patients is important because clinical intervention may be required before substantial respiratory deterioration occurs.

In routine clinical practice, however, anti-MDA5 antibody testing is not always immediately available (7). This is clinically relevant because treatment decisions often need to be made before serologic results are returned. Early management of DM-ILD is mainly guided by the patient’s clinical severity. In the present study, we used the first 48 hours after admission as a standardized baseline period for data collection, as information obtained during this early period may reflect the patient’s initial disease activity and respiratory reserve. Thus, a risk assessment approach based on data available within the first 48 hours may complement clinical assessment, particularly in settings where specialized serologic testing is not readily accessible.

Previous studies have identified a variety of prognostic indicators in DM-ILD, including clinical, serologic, radiologic, and functional parameters. However, many existing models rely on a limited set of predictors, often from a single domain, or on anti-MDA5 antibody status itself. This design may reduce their utility in the early admission setting, when serologic results are still pending (8). In addition, most prior studies have focused on mortality, short-term survival, or radiographic progression, whereas respiratory failure has been less well studied as a clinically relevant and actionable outcome (9). Since respiratory failure directly reflects disease worsening and may prompt treatment escalation, early prediction of this event may be especially significant (10).

High-resolution computed tomography (HRCT) plays a central role in evaluating ILD. In most previous studies, however, HRCT assessment has largely relied on visual interpretation or semiquantitative scoring by radiologists (11, 12). However, these approaches are inherently subjective and may fail to capture subtle imaging information that is not readily apparent (13, 14). Recent advances in machine learning have enabled automated extraction of high-dimensional imaging features from HRCT, offering a more objective characterization of pulmonary abnormalities (15, 16). Several studies have suggested that combining quantitative imaging features with clinical variables may improve risk stratification in inflammatory myopathies (9). Nevertheless, the value of such imaging-derived features for predicting respiratory failure at admission in patients with DM-ILD remains insufficiently explored, particularly when anti-MDA5 antibody results are unavailable at admission.

This study aimed to develop and internally test a multimodal model for predicting 90-day respiratory failure in patients with DM-ILD when anti-MDA5 antibody status was unavailable at admission. This outcome reflects early clinical worsening more directly than longer-term endpoints.

## 2 Methods

### 2.1 Patient population

**Figure 1:**
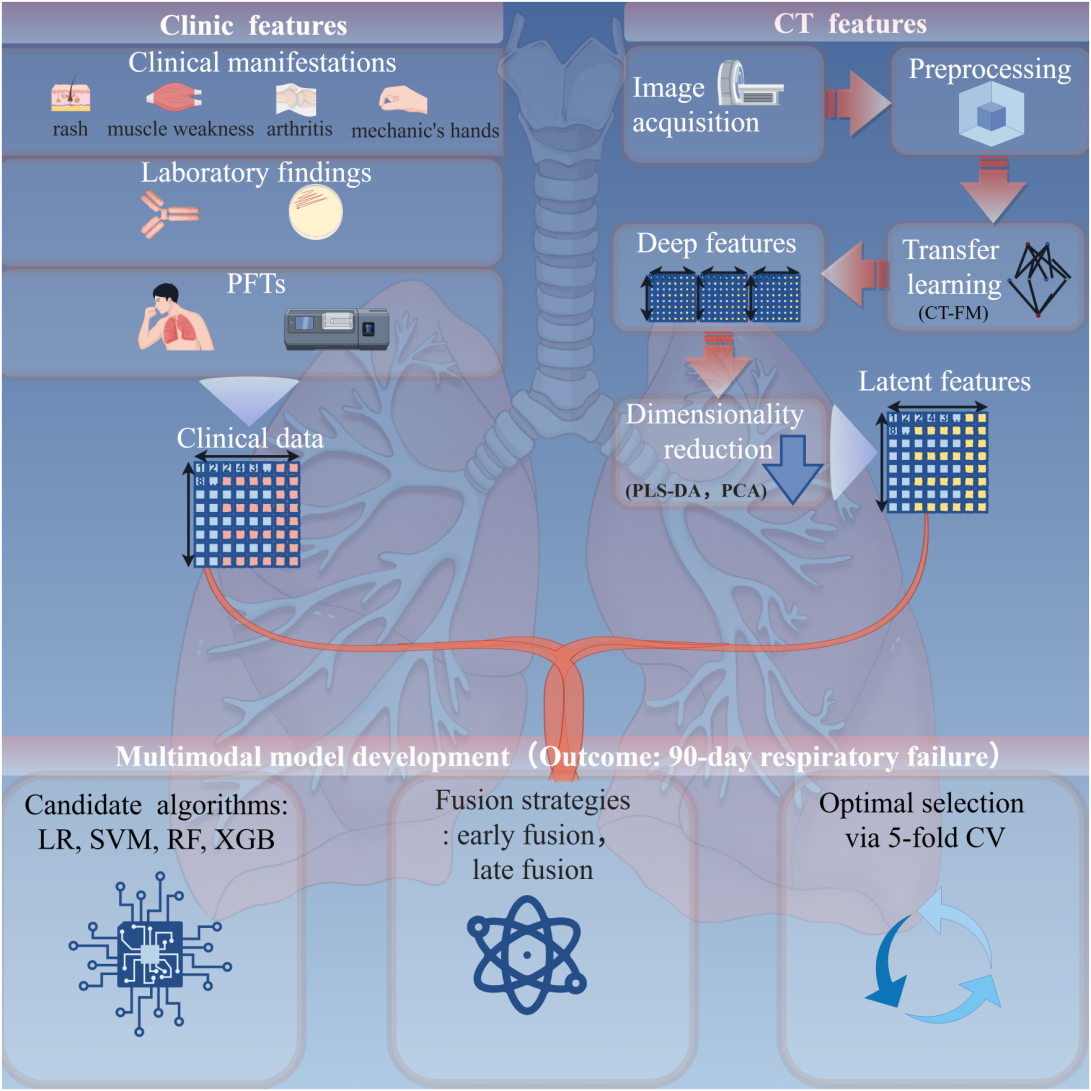
Overview of the study design and model development workflow. Clinical variables, laboratory findings, pulmonary function parameters, and HRCT scans collected within 48 hours of admission were used to develop the model. CT latent features were extracted using a pretrained CT-FM encoder, then reduced by PCA or PLS-DA, and combined with clinical data using early- and late-fusion strategies. The final model was selected using five-fold cross-validation for predicting 90-day respiratory failure. PFTs, pulmonary function tests; CT, computed tomography; CT-FM, vision foundation models for computed tomography; PCA, principal component analysis; PLS-DA, partial least squares discriminant analysis; LR, logistic regression; SVM, support vector machine; RF, random forest; XGB, XGBoost; CV, cross-validation; SHAP, SHapley Additive exPlanations.

This study was designed as a single-center retrospective analysis, taking place at the First Hospital of Lanzhou University. The patient population consisted of individuals who were consecutively admitted to our facility during the period from November 2016 to November 2025. No formal sample-size calculation was performed, the cohort included all eligible patients admitted during the study period. **Figure 1** provides an overview of the study design and model development workflow.

The identification of DM was determined based on widely recognized classification standards, which encompass the Bohan and Peter criteria, as well as the guidelines set forth by the European League Against Rheumatism/American College of Rheumatology (EULAR/ACR) classification criteria (1, 17, 18). The diagnosis of DM-ILD was confirmed in a multidisciplinary team that included rheumatologists, pulmonologists, and radiologists on the basis of clinical findings and HRCT features (19, 20). Additional investigations were performed when clinically indicated to exclude other potential causes of ILD.

**Figure 2:**
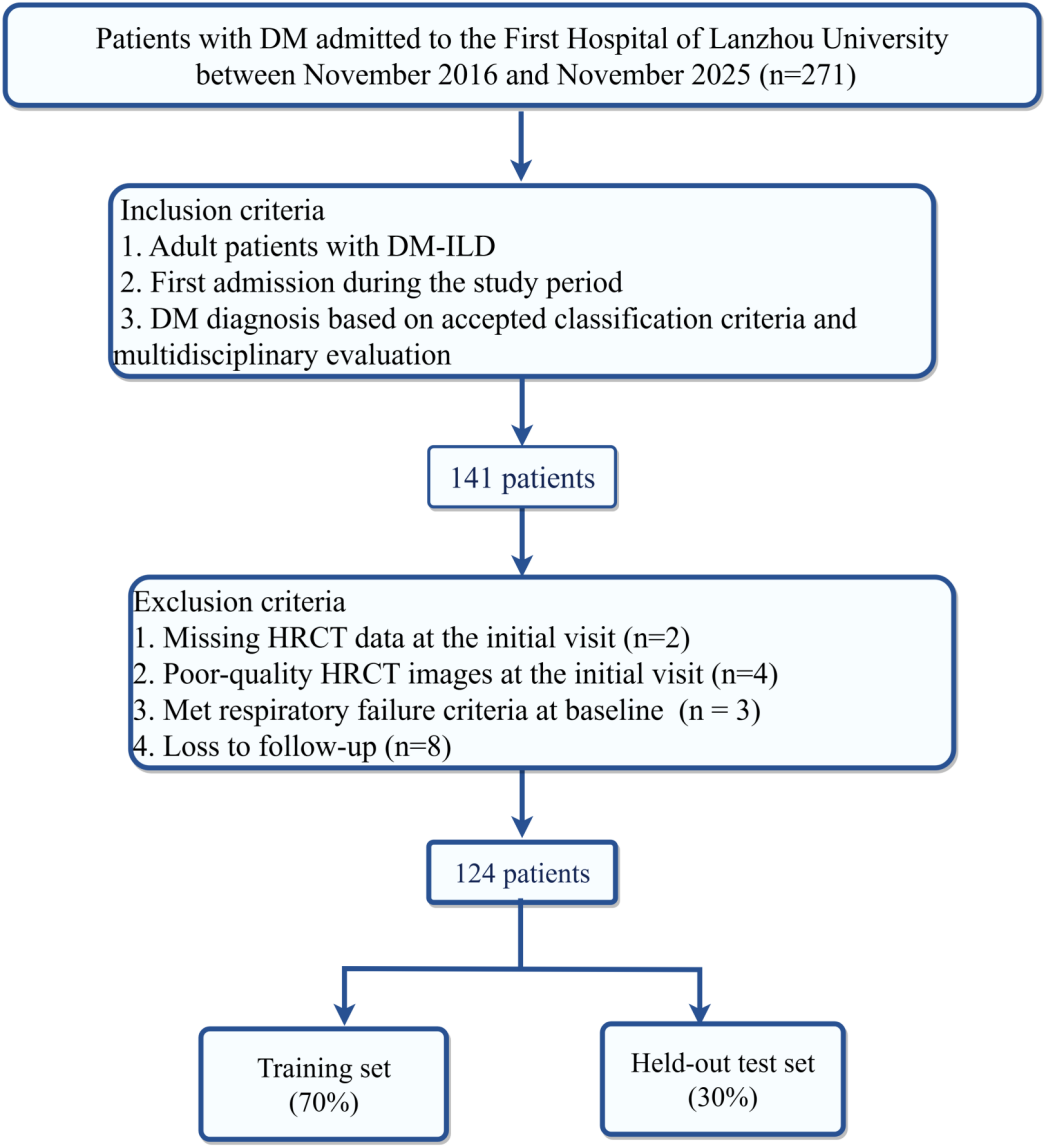
Flowchart of patient selection. A comprehensive screening was conducted involving 271 patients diagnosed with DM. After selection, 124 DM-ILD patients were selected for further analysis.

Our study included all patients who were 18 years of age or older and were admitted to our hospital for the first time throughout the duration of the study period. Index hospitalization was defined as the first admission to our institution during the study period. Baseline was defined as the first 48 hours after admission, and only variables collected during this period were considered candidate predictors. Patients were excluded if baseline HRCT images were unavailable or non-diagnostic, if respiratory failure was already present at baseline, or if follow-up data were incomplete. The study flowchart and final sample size are shown in **Figure 2**.

### 2.2 Outcome definition

The primary outcome was new-onset respiratory failure within 90 days after baseline, defined using a prespecified clinical adjudication framework. Respiratory failure was defined based on arterial blood gas measurements, specifically as an arterial oxygen partial pressure (PaO_2_) less than 60 mmHg, which may or may not be accompanied by an arterial carbon dioxide partial pressure (PaCO_2_) above 50 mmHg (21). This physiologic definition served as the primary criterion for outcome adjudication. For patients already receiving supplemental oxygen at the time of arterial blood gas testing, a prespecified adjunct rule was applied only when PaO_2_ values required by the primary outcome definition were unavailable. In these patients, outcome adjudication was based on the explicit diagnosis of respiratory failure documented in the medical records. Respiratory support records were reviewed as part of outcome ascertainment but were not used as an independent adjudication criterion. Outcome ascertainment was based on inpatient records, laboratory data, respiratory support records, outpatient follow-up records, and readmission data. Follow-up through January 1, 2026, ensured that all included patients had at least 90 days of observation. Only new-onset events occurring after the baseline window were counted as outcomes, and patients with respiratory failure already present at baseline were excluded. Outcome adjudication was performed independently by two clinicians, and disagreements were resolved by consensus.

### 2.3 HRCT acquisition

HRCT examinations were performed using Siemens SOMATOM Definition Flash dual-source CT scanners and SOMATOM Definition AS 64-slice CT scanners. The scans were conducted while the patients were positioned in a supine stance, ensuring that they were fully inhaling. In order to optimize the imaging process, both of the patients’ arms were elevated above their heads.

The detailed CT acquisition parameters are presented in **Supplementary Table 1**. Images were initially acquired with a slice thickness of 5 mm and reconstructed into 1-mm sections using a high-spatial-frequency algorithm. All deep learning analyses were performed using the 1-mm reconstructed images.

### 2.4 Deep latent features extraction via transfer learning

We employed vision foundation models for computed tomography (CT-FM), a large-scale 3D image-based pre-trained foundation model, as a fixed feature extractor (22). DICOM images were converted into 3D volumes and preprocessed in medical open network for AI (MONAI) by reorienting to standard superior-posterior-left (SPL) coordinates, linear scaling Hounsfield unit values from -1024 to 2048 to the 0 to 1 range with clipping, and foreground cropping using the body contour to remove background air. The same preprocessing pipeline was applied to all scans. Isotropic resampling was not performed because all HRCT images used for feature extraction were thin-section reconstructed images, and additional isotropic resampling would have introduced interpolation of voxel intensities and spatial texture patterns. This choice was also consistent with the CT-FM framework, which was developed using anisotropic CT preprocessing and has been reported to maintain robust embeddings across diverse spacing and acquisition settings. The study cohort did not undergo fine-tuning of the model, and the weights of the encoder stayed unchanged throughout the feature extraction process. Using 3D adaptive average pooling, the encoder generated multi-scale feature maps, which were then compressed into a fixed 512-dimensional latent feature vector for each 3D CT volume. When multiple CT series were available during the baseline period, the earliest thin-section inspiratory series within the baseline window was selected according to a predefined rule. Feature extraction was implemented in Python 3.9 using PyTorch and related libraries (22).

### 2.5 Variables and data preprocessing

Anti-MDA5 antibody status was not included in the predictor set because this test was not routinely available during the early admission period at our institution. Candidate predictors included demographic data, clinical manifestations, laboratory results, arterial blood gas measurements, pulmonary function parameters, and CT-derived latent features. The proportion of missing data for each predictor was summarized before model development. Missingness was low for most candidate predictors.

Using stratified sampling by outcome status, the dataset was randomly split into a training set (70%) and a held-out test set (30%). All preprocessing steps were derived exclusively from the training set and then directly applied to the held-out test set. Model development occurred solely within the training set. Categorical variables were one-hot encoded, each category becoming a separate binary feature. Missing continuous values were imputed with the training-set median. Missing categorical values were filled with prespecified clinically negative or normal categories, because normal or negative findings are often left undocumented in clinical records; missingness was therefore treated as normal or negative. Antinuclear antibody (ANA) pattern was left missing when undocumented, as this could not be assumed to indicate a normal or negative finding. No outcome information was used in any of these steps. Z-score scaling was used to standardize continuous variables.

To reduce information leakage, imputation, encoding, scaling, dimensionality reduction, and model fitting were performed within unified pipelines. During each iteration of the cross-validation process, the parameters for preprocessing were exclusively estimated using data from the training folds. Then, these parameters were applied to the corresponding validation fold. After model selection, the final pipeline was refitted using the entire training set and then applied unchanged to the held-out test set. **Supplementary Table 2** offers a detailed overview of the strategies for overfitting reduction.

### 2.6 Dimensionality reduction of deep latent CT features

Two approaches were used to reduce the dimensionality of the standardized CT latent features. Principal component analysis (PCA) was used as an unsupervised method, with the number of components selected as the minimum required to explain 95% of the total variance in the training set. Partial least squares discriminant analysis (PLS-DA) was used as a supervised method with the binary outcome as the response variable. The ideal number of latent components was established through the application of stratified five- fold cross-validation conducted in the training set (23, 24). Because PLS-DA is supervised, both model fitting and component selection were performed within the training data only.

### 2.7 Model development, architecture, and hyperparameter optimization

Because of the small cohort, we decided in advance which feature sets, dimension-reduction methods, and algorithms to test. Considering the presence of categorical variables, four prespecified machine learning algorithms were evaluated: logistic regression (LR), support vector machine (SVM), random forest (RF), and extreme gradient boosting (XGBoost) (25–28). Each algorithm was trained using five feature configurations: clinical features only (clinical model), PCA-reduced CT latent features only (CT model 1), PLS-DA-reduced CT latent features only (CT model 2), early fusion of clinical features with PCA-reduced CT latent features (Early-fusion model 1), and early fusion of clinical features with PLS-DA-reduced CT latent features (Early-fusion model 2). These feature configurations were compared to evaluate the relative contribution of clinical data, CT-derived latent features, and their combinations within a common modeling framework.

Hyperparameters were optimized within the training set using stratified five-fold cross-validation, with mean AUC as the primary selection metric. All model selection procedures, including hyperparameter tuning and feature-configuration comparison, were restricted to the training set. For comparative late-fusion analysis, the best-performing clinical-only model and the best-performing CT-only model, as determined by cross-validated AUC in the training set, were selected and combined by averaging their predicted probabilities. To address class imbalance, balanced class weights were applied in LR, SVM, and RF models. The scale_pos_weight parameter for XGBoost was configured (28). These imbalance-handling strategies were specified before model evaluation and applied only during training.

### 2.8 Model evaluation and interpretability

All final performance metrics were calculated in the held-out test set. The area under the receiver operating characteristic curve (AUROC) was used as the primary measure of model discrimination. The 95% confidence intervals (CIs) were estimated by bootstrap resampling with 1, 000 repetitions. The classification threshold was determined in the training set by maximizing the mean F1 score during stratified five-fold cross-validation. This thresholding strategy was employed solely for exploratory evaluation and should not be interpreted as a clinical decision threshold. For interpretability, standardized coefficients from penalized logistic regression and SHAP values from nonlinear models were examined.

### 2.9 Statistical analysis

The whole data analyses during our study were performed in Python 3.9 using XGBoost, PyTorch, MONAI, NumPy, scikit-learn, and pandas. Continuous variables are presented as mean ± standard deviation when normally distributed and as median with interquartile range when non-normally distributed. For continuous variables, we utilized either the independent-samples t test or the Mann-Whitney U test, depending on the distribution characteristics of the data. For categorical variables, we applied the chi-square test or Fisher’s exact test to assess relationships between groups. Statistical analyses were carried out as two-sided tests, ensuring that we examined the possibility of differences in both directions. A P value of less than 0.05 was deemed statistically significant.

## 3 Results

### 3.1 Study population and baseline characteristics

The final analysis comprised 124 patients with DM-ILD. Of these, 26 (21%) developed respiratory failure within 90 days after baseline, whereas 98 (79%) did not. **Table 1** summarizes baseline demographic, clinical, laboratory, immunologic, and pulmonary function characteristics by respiratory failure status. In our cohort, most of the patients were female. Compared with patients without respiratory failure, those who developed respiratory failure had a higher prevalence of periungual erythema, arthritis, and mechanic’s hand. They also had lower albumin and C3 levels, higher ESR and serum ferritin levels, and worse pulmonary function at baseline, including lower FVC% predicted, FEV1% predicted, and DLCO% predicted. Other baseline characteristics are presented in **Table 1**.

**Table 1:** Baseline characteristics of study participants.

Characteristic	All patients	Patients without RF	Patients with RF	P value
	(n=124)	(n=98)	(n=26)	
Age (years)	53.00 (44.00, 59.25)	52.00 (44.00, 59.75)	54.00 (51.00, 57.75)	0.495
Sex Female	95 (76.6%)	76 (77.6%)	19 (73.1%)	0.827
Male	29 (23.4%)	22 (22.4%)	7 (26.9%)	
Muscle weakness	67 (54.0%)	51 (52.0%)	16 (61.5%)	0.521
Rash	94 (75.8%)	70 (71.4%)	24 (92.3%)	0.051
Periungual erythema	11 (8.9%)	5 (5.1%)	6 (23.1%)	0.011
Arthritis	33 (26.6%)	15 (15.3%)	18 (69.2%)	<0.001
Mechanic’s hand	11 (8.9%)	5 (5.1%)	6 (23.1%)	0.011
Skin ulcer	11 (8.9%)	8 (8.2%)	3 (11.5%)	0.698
ALT (U/L)	50.50 (25.00, 107.75)	47.00 (24.25, 94.25)	68.00 (36.75, 222.50)	0.046
AST (U/L)	58.50 (30.00, 142.00)	57.50 (29.25, 139.00)	86.50 (43.25, 215.25)	0.141
AST/ALT	1.58 (1.24, 2.20)	1.55 (1.23, 2.19)	1.65 (1.31, 2.29)	0.637
ALB (g/L)	33.28 ± 5.09	34.13 ± 4.95	30.08 ± 4.34	<0.001
ALB/GLB	1.19 ± 0.32	1.21 ± 0.32	1.12 ± 0.30	0.181
PA (mg/L)	170.44 ± 78.75	176.36 ± 78.69	144.40 ± 77.56	0.261
CK (U/L)	173.00 (58.00, 1836.00)	190.00 (59.00, 1873.00)	138.00 (48.00, 1655.50)	0.995
CK-MB (U/L)	22.00 (15.00, 79.00)	20.00 (14.00, 58.00)	78.50 (27.50, 227.50)	<0.001
LDH (U/L)	392.00 (291.00, 547.50)	386.00 (282.00, 536.00)	444.50 (327.50, 989.50)	0.056
Creatinine (umol/L)	55.50 (47.73, 63.92)	55.65 (48.48, 64.88)	55.50 (45.65, 61.77)	0.393
Urea/Crea	122.00 (94.00, 171.50)	117.00 (91.00, 163.00)	148.50 (111.25, 185.00)	0.015
ESR (mm/h)	27.00 (14.00, 42.00)	25.00 (14.25, 37.75)	35.00 (15.00, 56.75)	0.046
PCT (ng/mL)	0.07 (0.04, 0.12)	0.07 (0.04, 0.12)	0.05 (0.04, 0.09)	0.405
Ferritin (ng/mL)	416.00 (181.82, 820.50)	355.50 (112.75, 657.50)	706.00 (375.50, 1085.25)	0.001
IgG (g/L)	13.30 (11.05, 17.73)	13.30 (11.15, 17.82)	12.65 (11.42, 13.88)	0.573
IgA (g/L)	2.45 (1.84, 3.10)	2.50 (1.86, 3.11)	2.07 (1.95, 2.19)	0.481
IgM (g/L)	1.53 (0.95, 2.36)	1.53 (0.93, 2.36)	3.72 (2.58, 4.85)	0.298
C3 (g/L)	0.93 ± 0.27	0.96 ± 0.27	0.84 ± 0.25	0.043
C4 (g/L)	0.22 (0.17, 0.28)	0.22 (0.17, 0.28)	0.21 (0.17, 0.23)	0.384
Anti-Jo-1 antibody (1:100)	Positive: 12 (12.5%)	Positive: 9(11.0%)	Positive: 3 (14.3%)	0.635
Anti-Ro-52 antibody (1:100)	Positive: 74 (66.1%)	Positive: 60 (66.7%)	Positive: 14 (63.6%)	0.086
Anti-M2 antibody (1:100)	Positive: 19 (17.0%)	Positive: 17 (18.9%)	Positive: 2 (9%)	0.343
ANA antibody titer(1:100)	Positive: 57 (50.4%)	Positive: 44(48.4%)	Positive: 13(59%)	0.566
ANA pattern (nuclear)	AC-3: 32 (69.6%)	AC-3: 26 (70.3%)	AC-3: 6 (66.7%)	0.772
ANA pattern (cytoplasmic)	AC-19:38 (90.5%)	AC-19: 28 (90.3%)	AC-19: 10 (90.9%)	0.592
aCL IgG (GPL/mL)	5.40 (3.10, 9.70)	5.99 (3.96, 10.27)	3.56 (2.07, 7.36)	0.026
aβ2GPI IgG (U/mL)	4.04 (2.09, 9.61)	3.62 (2.88, 8.46)	6.42 (1.69, 10.15)	1
SaO2	93.00 (90.00, 95.00)	94.00 (90.25, 95.75)	93.00 (81.00, 93.25)	0.329
FVC%predicted	67.18 ± 9.73	69.84 ± 7.89	58.01 ± 10.03	<0.001
FEV1%predicted	64.47 (57.92, 70.93)	68.56 (61.21, 71.76)	53.90 (49.70, 62.25)	<0.001
FEV1/FVC ratio	0.85 ± 0.03	0.85 ± 0.03	0.87 ± 0.03	0.006
DLCO%predicted	56.00 (50.20, 61.40)	58.80 (52.45, 62.40)	45.80 (44.98, 50.33)	<0.001

Data are presented as mean ± standard deviation, median (interquartile range), or n (%), as appropriate. RF indicates respiratory failure within 90 days after baseline. ALT, alanine aminotransferase; AST, aspartate aminotransferase; ALB, albumin; GLB, globulin; PA, prealbumin; CK, creatine kinase; CK-MB, creatine kinase-MB; LDH, lactate dehydrogenase; ESR, erythrocyte sedimentation rate; PCT, procalcitonin; Ig, immunoglobulin; ANA, antinuclear antibody; aCL, anticardiolipin antibody; aβ2GPI, anti-beta-2 glycoprotein I antibody; SaO2, arterial oxygen saturation; FVC, forced vital capacity; FEV1, forced expiratory volume in 1 second; DLCO, diffusing capacity of the lung for carbon monoxide.ANA patterns were classified by ICAP nomenclature, AC-3, nuclear fine speckled; AC-19, cytoplasmic speckled.

### 3.2 Model performance of all candidate models

**Figure 3:**
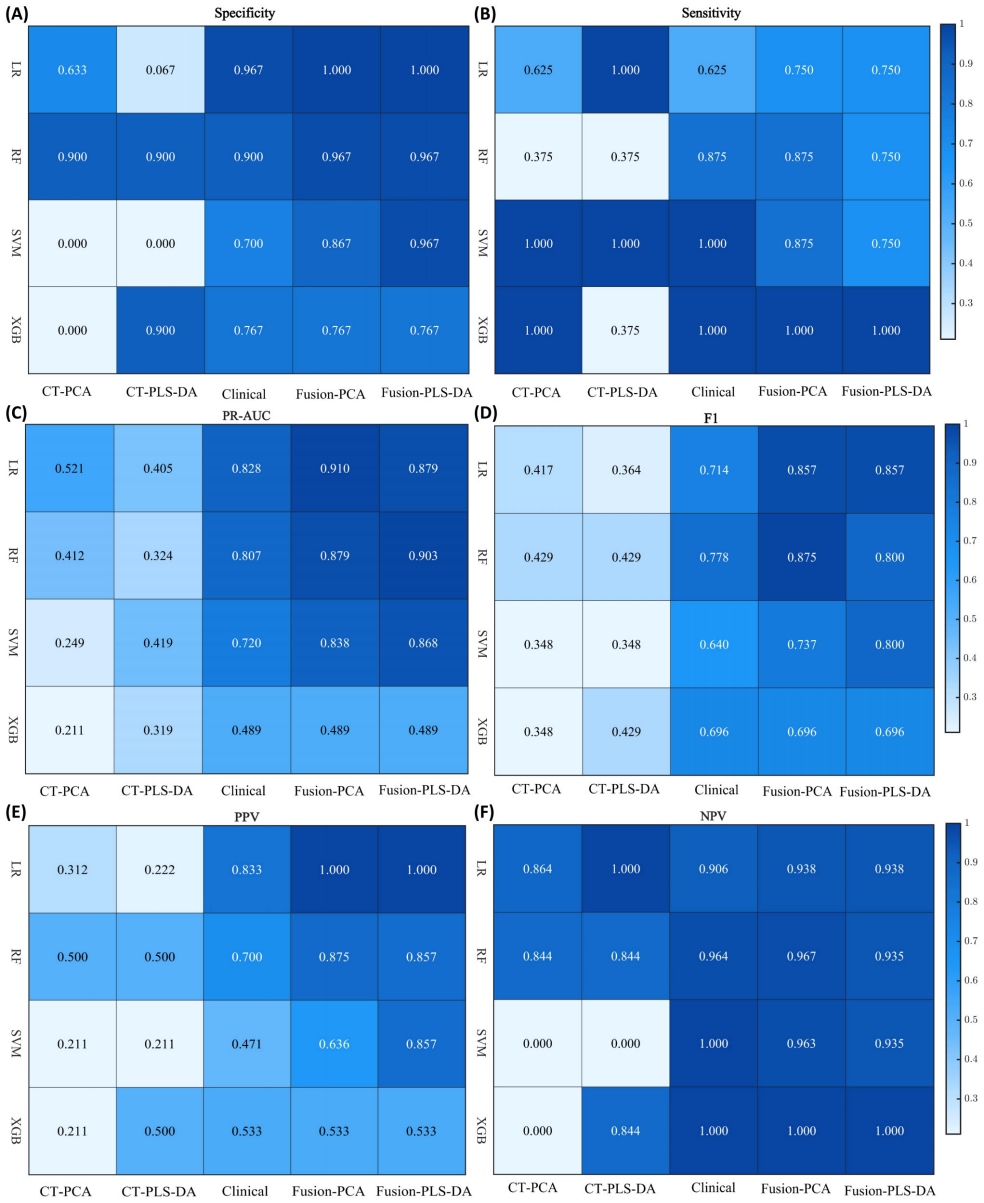
Model performance in the held-out test set. Heatmaps of **(A)** specificity, **(B)** sensitivity, **(C)** PR-AUC, **(D)** F1 score, **(E)** PPV, and **(F)** NPV for LR, RF, SVM, and XGBoost cross five feature sets: CT-PCA, CT-PLS-DA, Clinical, Fusion-PCA, and Fusion-PLS-DA. Overall, models combining CT and clinical features performed better than CT-only models.

Overall, fusion models performed better than CT-only models and generally showed better discrimination than single-modality approaches in both ROC and precision-recall analyses. CT-only models had limited predictive value when used alone, whereas combining CT latent features with clinical and functional variables consistently improved model performance. Therefore, the higher performance of the fusion models may mainly reflect the strong signals from early clinical and functional data. Similar patterns were observed in the heatmaps and radar plots. The metrics for the performance of each candidate model can be found in **Supplementary Table 3**, and the selected representative models are shown in **Figure 3**.

Among single-modality models, the clinical RF model performed best, while CT-only models showed limited predictive value. The fusion models that showed the highest effectiveness in this research were centered around RF algorithms. Specifically, the early-fusion PCA-based RF model achieved the highest overall performance in the held-out test set. The late-fusion model performed well but did not surpass the top early-fusion models.

### 3.3 The selection of the final model

**Figure 4:**
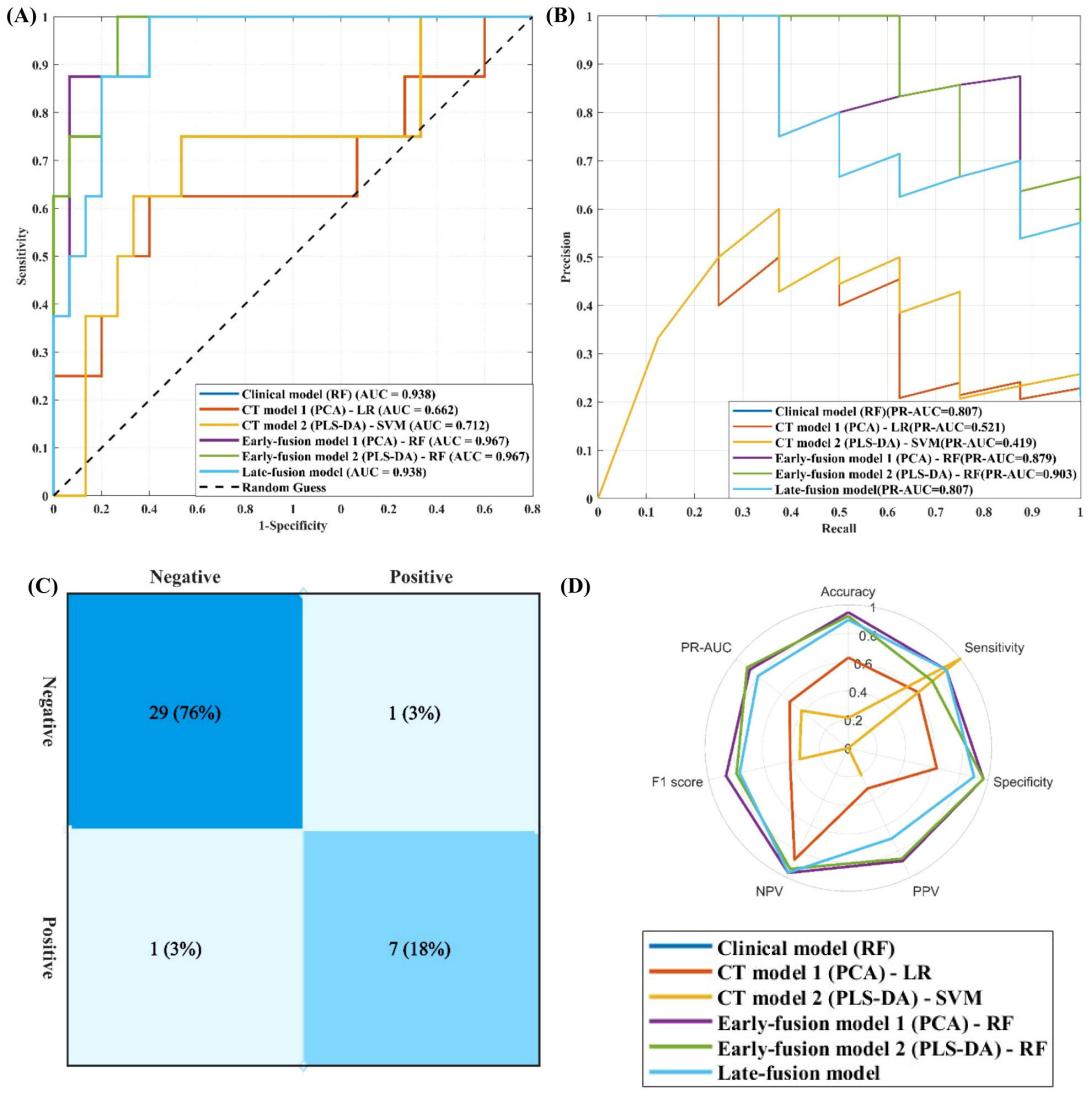
Performance comparison of selected models in the held-out test set. **(A)** ROC curves for clinical model, CT-only models, early-fusion models, and the late-fusion model. **(B)** Precision-recall curves showing each model’s ability to identify positive cases. **(C)** Confusion matrix of the final early-fusion PCA-based RF model. **(D)** Radar plot summarizing multiple performance metrics.

We selected the early-fusion PCA-based RF model using five-fold cross-validation in the training set. We then tested this model once in the held-out test set. The model achieved strong performance: AUC 0.967, PR-AUC 0.879, accuracy 0.947, sensitivity 0.875, specificity 0.967. Detailed results of all selected models are provided in **Table 2**. In the held-out test set, the model yielded few misclassifications. However, because the number of respiratory failure events in the test set was limited, these metrics should be interpreted cautiously (**Figure 4**). Additional stability analyses, including multiple random train-test splits, repeated 5-fold cross-validation, and bootstrap optimism correction (0.632+ method), are summarized in **Supplementary Table 4**. The optimism-corrected AUC for the final model was 0.905, indicating good discrimination after accounting for overfitting.

**Table 2:** Comparative performance of selected representative models in the held-out test set.

Model	AUC (95% CI)	Accuracy	Sensitivity	Specificity	PPV	NPV	F1 score	PR-AUC	Brier score	H-L *P* value
Clinical model (RF)	0.938 (0.848-1.000)	0.895	0.875	0.900	0.700	0.964	0.778	0.807	0.116	0.850
CT model 1 (PCA) - LR	0.662 (0.383-0.911)	0.632	0.625	0.633	0.312	0.864	0.417	0.521	0.252	0.310
CT model 2 (PLS-DA) - SVM	0.712 (0.475-0.919)	0.211	1.000	0.000	0.211	0.000	0.348	0.419	0.169	0.509
Early-fusion model 1 (PCA) - RF	0.967 (0.899-1.000)	0.947	0.875	0.967	0.875	0.967	0.875	0.879	0.105	0.570
Early-fusion model 2 (PLS-DA) - RF	0.967 (0.900-1.000)	0.921	0.750	0.967	0.857	0.935	0.800	0.903	0.109	0.689
Late-fusion model	0.938 (0.848-1.000)	0.895	0.875	0.900	0.700	0.964	0.778	0.807	0.118	0.773

Performance metrics were calculated in the held-out test set. RF, random forest; LR, logistic regression; SVM, support vector machine; PLS-DA, partial least squares discriminant analysis; PCA, principal component analysis; PR-AUC, area under the precision-recall curve; AUC, area under the receiver operating characteristic curve; NPV, negative predictive value; PPV, positive predictive value; CI, confidence interval; H-L: Hosmer–Lemesho.

### 3.4 Model interpretability

**Figure 5:**
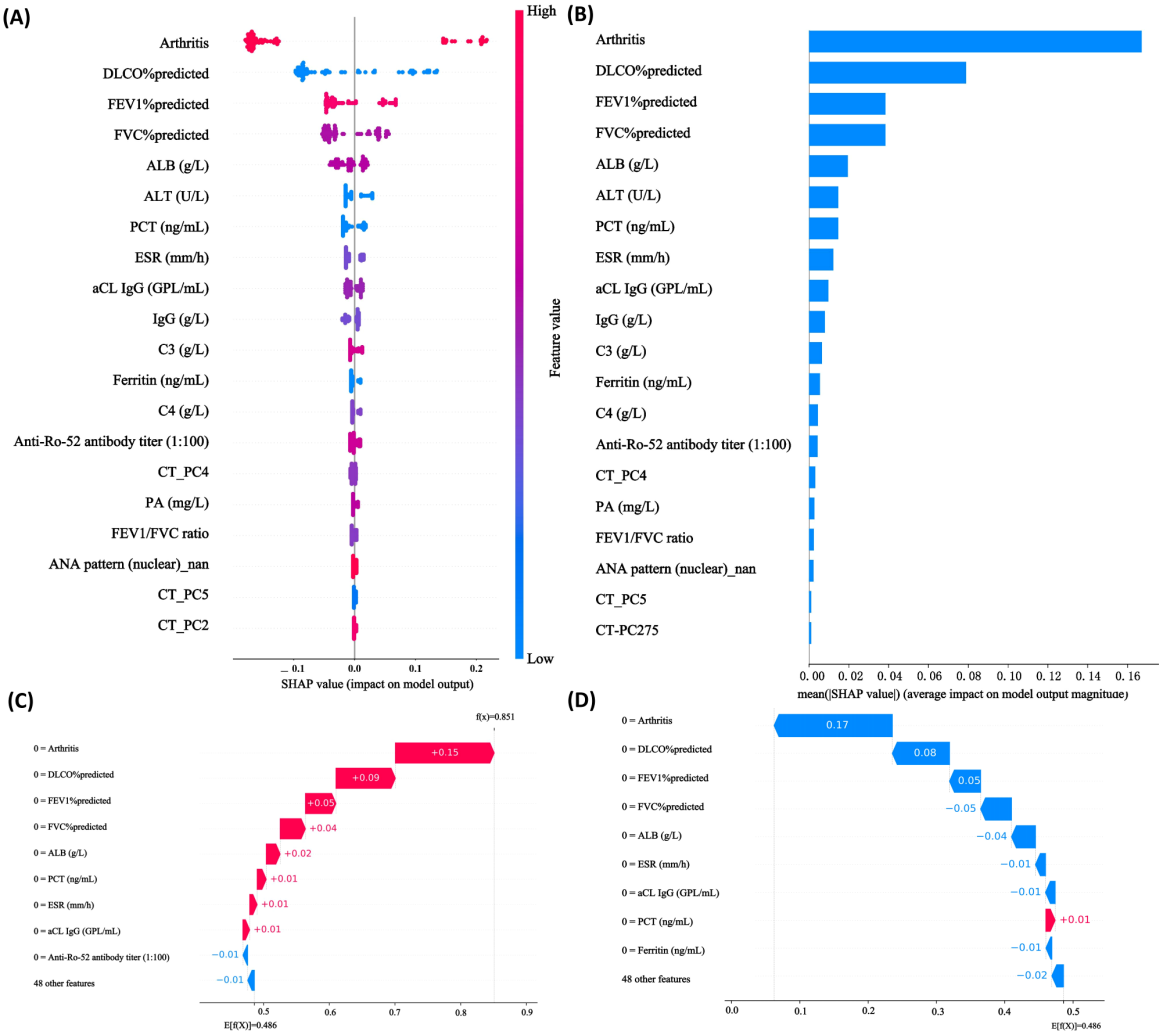
Feature contributions. **(A)** A SHAP summary plot illustrating every feature’s effect on the model output. **(B)** SHAP Bar Plot indicating the overall importance of the top features. **(C, D)** SHAP waterfall plots for a positive prediction and a negative prediction.ALB: albumin; ALT: alanine aminotransferase; ESR: erythrocyte sedimentation rate; PCT: procalcitonin; aCL IgG: anti-cardiolipin IgG; Anti-Ro-52: anti-Ro-52 antibody titer; DLCO% predicted: percent predicted diffusing capacity for carbon monoxide; FEV1% predicted: percent predicted forced expiratory volume in 1 second; FVC% predicted: percent predicted forced vital capacity; FEV1/FVC ratio: ratio of FEV1 to FVC; CT_PC2, CT_PC4, CT_PC5, CT_PC275: principal components derived from CT latent features; SHAP: SHapley Additive exPlanations.

SHAP values were used to show feature importance. Arthritis was the most important predictor in the final model. Pulmonary function variables, including DLCO% predicted, FEV1% predicted, and FVC% predicted, also contributed substantially. In addition, several laboratory variables influenced model output, including albumin, ALT, PCT, ESR, aCL IgG, IgG, C3, and ferritin. CT-derived latent features, including CT_PC4, CT_PC5, CT_PC2, and CT_PC275, also ranked among the influential predictors, indicating that they contributed to model performance in this cohort (**Figures 5A, B**). The SHAP dependence and interaction plots further illustrated how key features contributed to the model output (**Figures 5C, D**).

## 4 Discussion

In this single-center retrospective study, a multimodal model was developed and tested to predict 90-day respiratory failure in patients with DM-ILD, particularly for regions where anti-MDA5 antibody testing is not routinely available. Such a model has practical significance, as it allows for early identification of high-risk patients using readily accessible clinical and laboratory indicators, facilitating timely intervention, closer monitoring, and potentially improved outcomes in resource-limited settings. Among the candidate models, the early-fusion PCA-based RF model showed the most favorable performance. These findings suggest that early admission-based risk assessment may be feasible before anti-MDA5 antibody results become available.

The final model performed so well largely because baseline lung function and admission lab markers are already strongly predictive on their own. These variables are closely tied to short-term respiratory decline and likely account for a big part of the model’s performance. So the model is better seen as an early severity-stratification tool based on admission data, not as proof that CT-derived latent features alone can predict respiratory failure. Baseline respiratory variables should be interpreted as early indicators of vulnerability to subsequent respiratory deterioration, rather than as evidence that the model predicts respiratory failure independently of early respiratory impairment.

From a clinical perspective, arthritis emerged as an important predictor of subsequent respiratory failure. Although arthritis is an extrapulmonary manifestation of DM, it may also indicate a higher inflammatory burden at presentation (29, 30). Research on idiopathic inflammatory myopathies indicates a correlation between musculoskeletal involvement and increased immune activation, including cytokine dysregulation and type I interferon pathways, both of which are implicated in ILD progression (31, 32). In this cohort, arthritis may reflect a higher-risk inflammatory presentation at admission. This interpretation is supported by our findings that arthritis, as well as periungual erythema and mechanic’s hands, were more frequent in patients who later developed respiratory failure. Together, these features may mark a higher-risk clinical presentation at admission (3, 33). In our multivariable model, which adjusted for inflammatory markers, autoantibodies, and baseline pulmonary severity, arthritis continued to emerge as the most prominent predictor. Arthritis should be interpreted cautiously as a possible marker of a high-risk clinical phenotype, rather than as a causal factor.

Pulmonary function was also closely associated with later respiratory failure, indicating that impaired baseline respiratory reserve is a key determinant of short-term decompensation (34). Lower FVC% predicted, FEV1% predicted, and DLCO% predicted all reflect reduced capacity to tolerate additional pulmonary injury (35, 36). Among these, DLCO is particularly informative as it reflects impairment in alveolar-capillary gas exchange and may indicate early interstitial damage or microvascular involvement (37, 38). Reduced FVC is consistent with restrictive ventilatory impairment due to decreased lung compliance and more extensive parenchymal involvement (35, 39). Importantly, pulmonary function testing is relatively inexpensive, widely available, and easy to perform in routine clinical settings. Compared with specialized serologic assays, which may require external processing and delay clinical decision-making, pulmonary function parameters can usually be obtained promptly after admission. From both a practical and economic perspective, reliance on readily accessible functional measures may reduce delays in risk stratification and alleviate patient burden, supporting their utility in admission-based risk stratification. In this setting, their main value lies in early availability and prognostic signal at admission.

CT-derived latent features appeared to provide complementary information, but their independent predictive value was limited in this cohort. Their contribution was most apparent when they were combined with clinical, laboratory, and pulmonary function variables. HRCT captures the extent and distribution of lung injury, but visual assessment is inherently subjective and may miss subtle imaging patterns (40, 41). In contrast, deep learning-based features may capture imaging patterns not fully represented by conventional visual assessment (42–44). However, respiratory failure cannot be predicted only based on the extent of apparent lung injury (30, 45). Rather, it reflects the interaction between pulmonary structural damage and systemic processes such as inflammation, immune dysregulation, and extrapulmonary disease activity (30, 45, 46). This likely explains why imaging features alone showed limited predictive value but became more informative in a multimodal setting.

In addition, several laboratory variables were identified as useful predictors of respiratory failure. The overall pattern suggests that adverse pulmonary outcomes are closely linked to systemic disease activity (29). Emerging evidence indicates robust deposition of immunoglobulins and the complement component in the alveolar epithelium of patients with DM-ILD. Reduced C3 levels may therefore suggest complement consumption and immune activation (3, 47). In DM, hyperferritinemia may not only correlate with disease severity but also be a biomarker for stratifying patients with elevated risk. Elevated ESR and ferritin levels indicate systemic inflammation, and hyperferritinemia has been associated with severe inflammatory states in DM-ILD (48–50). Meanwhile, Recent retrospective studies indicate that liver dysfunction is common among patients with DM (51, 52). Increased ALT and CK-MB levels may indicate hepatic and myocardial involvement, and a higher urea/creatinine ratio may reflect catabolic stress or impaired perfusion (53–55). Hypoalbuminemia often reflects a state of protein-energy malnutrition, excessive catabolic consumption, or impaired synthesis due to systemic inflammation (56, 57). Low albumin levels, heightened inflammatory activity, liver dysfunction, and immune dysregulation should not be viewed as isolated abnormalities. Persistent immune and inflammatory activation may promote catabolism and impair protein synthesis, thereby contributing to hypoalbuminemia, while hepatic injury may further weaken synthetic capacity and amplify the systemic inflammatory burden (51, 56, 58). Together, these abnormalities suggest that some patients were already experiencing inflammation, nutritional deficits, and multi-organ stress upon admission. This also highlights the need for a combined management approach early in the disease course. In newly diagnosed patients, timely nutritional support may help improve metabolic reserve and overall tolerance to illness. At the same time, active control of systemic inflammation and correction of immune dysregulation may be equally important, as these processes likely contribute to both pulmonary deterioration and extra-pulmonary organ involvement (29, 48, 50).

Methodologically, our findings suggest that multimodal integration may be useful in this setting. Compared with the corresponding single-modality models evaluated in this study, models combining clinical and imaging information showed better performance in the current cohort. However, this advantage should be interpreted within the present study design because of the sample size. Notably, anti-MDA5 antibody status was excluded to reflect a common real-world scenario in which results are not available early. We also used a transfer learning-based approach to extract high-dimensional CT features and applied dimensionality reduction before integrating them with structured data (14, 59, 60).

The classification profile of the final model provides additional information on its internal test-set performance. In the held-out test set, the model yielded few false negatives while maintaining high specificity. These counts should be interpreted cautiously because the test set was small. Although external validation is still needed, the model may assist early risk assessment during hospitalization.

This study has several notable strengths (1): it was conducted in a clinically realistic setting, focusing on patients whose anti-MDA5 antibody status was unavailable at admission; (2) it used respiratory failure as a clinically relevant and actionable outcome; (3) it integrated clinical, functional, laboratory, and imaging data; (4) it included a direct comparison between early-fusion and a comparator late-fusion approach; (5) CT features were extracted using a pretrained model and reduced in dimension before modeling; and (6) all predictors were obtained within the first 48 hours of admission, supporting early risk stratification.

A number of limitations must also be recognized. This research was a retrospective study conducted at a single center, featuring a sample size that is quite limited. Several measures were used to reduce bias, including consecutive screening, predefined eligibility criteria, standardized baseline data collection, prespecified missing-data handling, and independent outcome adjudication. Residual bias and center-specific practice patterns may still remain because of the retrospective single-center design. Multiple candidate pipelines were compared in a small dataset, so the reported performance may still be optimistic. Although model selection was restricted to the training set, testing several methods may still have introduced some bias. Therefore, the high AUC should be interpreted cautiously. Model selection and tuning were done with five-fold cross-validation in the training set. The final internal test used one held-out test set, so the results may still depend on the specific train-test split. Some baseline variables may have been affected by treatment started during the first 48 hours after admission. Because anti-MDA5 antibody testing was not routinely available at our center, we could not reliably assess its incremental prognostic value. Future multicenter studies with routine anti-MDA5 testing are needed. In addition, some variables may not be available in all acutely ill patients, and CT latent features remain less interpretable than conventional imaging descriptors. Although anti-MDA5 antibody testing was excluded to reflect delayed specialized serologic availability, the proposed model still requires HRCT, pulmonary function testing, CT-FM-based feature extraction, and computational support, which may limit implementation in centers without these resources. Finally, retrospective outcome assessment may have introduced some uncertainty. In particular, the pragmatic outcome definition, which incorporated both arterial blood gas criteria and clinical indicators in selected patients, may have introduced additional heterogeneity in adjudication. In a sensitivity analysis restricted to the 13 events that strictly met the prespecified ABG criterion, model performance showed a moderate reduction (AUC 0.85 vs. 0.97 in the primary analysis). This is likely due to the smaller sample size and the exclusion of some clinically overt cases. Nevertheless, our model still retained reasonable discriminative ability, suggesting that the heterogeneity in outcome definition did not undermine the overall findings (**Supplementary Table 5**). Our findings should therefore be interpreted as exploratory evidence from internal testing, and external validation is required before the model can be considered for clinical use. Further work should focus on external validation, prospective assessment, and the incorporation of longitudinal data, which will be essential to translate such models into reliable clinical practice.

## Data Availability

The raw data supporting the conclusions of this article will be made available by the authors, without undue reservation.
