# Supplementary material for: A transfer learning-based multimodal model for early prediction of 90-day respiratory failure in dermatomyositis-associated interstitial lung disease
Source: Front Immunol. 2026 Jul 16;17:1867606. doi: 10.3389/fimmu.2026.1867606 (PMC13422525; doi:10.3389/fimmu.2026.1867606)
Supplement: Supplementary file 1 [file Table1.docx]

**Supplementary Table 1.** CT Acquisition Parameters

| **Parameter** | **Value** |
| --- | --- |
| Tube voltage | 100–120 kV |
| Tube current modulation | CARE Dose4D |
| Collimation | 64 × 0.625 mm |
| Gantry rotation time | 0.35 s/rot |
| Pitch | 1.0 |
| Matrix size | 512 × 512 |
| Field of view | 250–350 mm |

This table summarizes the strategies used to mitigate overfitting in the primary modeling framework during model development and validation, including data partitioning, leakage-free preprocessing, dimensionality reduction, model regularization, cross-validation, class weighting, and bootstrap-based statistical inference. Sensitivity analyses that were not part of the primary modeling framework are not included in this table. L2 = ridge penalty.
