# Supplementary material for: A transfer learning-based multimodal model for early prediction of 90-day respiratory failure in dermatomyositis-associated interstitial lung disease
Source: Front Immunol. 2026 Jul 16;17:1867606. doi: 10.3389/fimmu.2026.1867606 (PMC13422525; doi:10.3389/fimmu.2026.1867606)
Supplement: Supplementary file 2 [file Table2.docx]

**Supplementary Table 2**. Strategies implemented to reduce overfitting and improve reproducibility

| **Methods** | | **Strategy** |
| --- | --- | --- |
| Internal held-out test set | | A 7:3 train-test split was used, and the held-out test set was used only for final internal evaluation. |
| Reproducibility control | | A fixed random seed, specifically set to 42, was utilized throughout various stages of the analysis. |
| Dimensionality reduction | PCA | PCA-based noise control was used, with 95% cumulative variance retained to reduce redundant information and feature dimensionality. |
|  | PLS-DA | Supervised PLS-DA was used to extract latent variables associated with the outcome, with the number of latent variables constrained to control model complexity. |
| Data source statement | | This study used in-hospital CT data that have not been publicly released; therefore, there was no risk of public-data-level information leakage. |
| Algorithm | LR | L2 regularization was applied, and the penalty parameter C was tuned within a constrained range to suppress overfitting. |
|  | SVM | An SVM utilizing a constrained penalty function was employed, and the range of parameters was limited to minimize the flexibility of the model. |
|  | RF | The complexity of the model was managed by limiting the depth of the trees, setting a minimum number of samples needed for a split, and specifying the minimum sample count in the leaf nodes. |
|  | XGBoost | A low learning rate, shallow trees, and L1/L2 regularization were used to improve model stability. |
| Class imbalance handling | | Balanced class weights or corresponding algorithm-specific parameters were used to reduce bias toward the majority class. |
| Statistical robustness assessment | | The 95% confidence interval of the AUC was estimated using 1,000 bootstrap resamples to quantify the uncertainty of model performance. |
