# Supplementary material for: A transfer learning-based multimodal model for early prediction of 90-day respiratory failure in dermatomyositis-associated interstitial lung disease
Source: Front Immunol. 2026 Jul 16;17:1867606. doi: 10.3389/fimmu.2026.1867606 (PMC13422525; doi:10.3389/fimmu.2026.1867606)
Supplement: Supplementary file 3 [file Table3.docx]

**Supplementary Table 3.** Performance of all candidate models

| **FeatureSet** | **Model** | **AUC (95% CI)** | **Accuracy** | **Sensitivity** | **Specificity** | **PPV** | **NPV** | **F1 score** | **PR-AUC** | **Brier score** | **H-L*P* value** | **Optimal threshold** |
| --- | --- | --- | --- | --- | --- | --- | --- | --- | --- | --- | --- | --- |
| Clinical | logistic regression | 0.929 (0.834-1.000) | 0.895 | 0.625 | 0.967 | 0.833 | 0.906 | 0.714 | 0.828 | 0.149 | 0.387 | 0.610 |
| Clinical | support vector machine | 0.875 (0.733-0.981) | 0.763 | 1.000 | 0.700 | 0.471 | 1.000 | 0.640 | 0.720 | 0.106 | 0.746 | 0.200 |
| Clinical | random forest | 0.938 (0.848-1.000) | 0.895 | 0.875 | 0.900 | 0.700 | 0.964 | 0.778 | 0.807 | 0.116 | 0.850 | 0.580 |
| Clinical | XGBoost | 0.858 (0.743-0.954) | 0.816 | 1.000 | 0.767 | 0.533 | 1.000 | 0.696 | 0.489 | 0.232 | 0.189 | 0.490 |
| CT_PCA | logistic regression | 0.662 (0.383-0.911) | 0.632 | 0.625 | 0.633 | 0.312 | 0.864 | 0.417 | 0.521 | 0.252 | 0.310 | 0.530 |
| CT_PCA | support vector machine | 0.458 (0.175-0.739) | 0.211 | 1.000 | 0.000 | 0.211 | 0.000 | 0.348 | 0.249 | 0.185 | 0.004 | 0.000 |
| CT_PCA | random forest | 0.627 (0.397-0.831) | 0.789 | 0.375 | 0.900 | 0.500 | 0.844 | 0.429 | 0.412 | 0.203 | 0.351 | 0.480 |
| CT_PCA | XGBoost | 0.500 (0.500-0.500) | 0.211 | 1.000 | 0.000 | 0.211 | 0.000 | 0.348 | 0.211 | 0.250 | 0.606 | 0.000 |
| CT_PLSDA | logistic regression | 0.546 (0.255-0.843) | 0.263 | 1.000 | 0.067 | 0.222 | 1.000 | 0.364 | 0.405 | 0.275 | 0.187 | 0.160 |
| CT_PLSDA | support vector machine | 0.712 (0.475-0.919) | 0.211 | 1.000 | 0.000 | 0.211 | 0.000 | 0.348 | 0.419 | 0.169 | 0.509 | 0.000 |
| CT_PLSDA | random forest | 0.554 (0.268-0.835) | 0.789 | 0.375 | 0.900 | 0.500 | 0.844 | 0.429 | 0.324 | 0.239 | 0.169 | 0.500 |
| CT_PLSDA | XGBoost | 0.638 (0.456-0.842) | 0.789 | 0.375 | 0.900 | 0.500 | 0.844 | 0.429 | 0.319 | 0.250 | 0.479 | 0.500 |
| Fusion_PCA | logistic regression | 0.958 (0.885-1.000) | 0.947 | 0.750 | 1.000 | 1.000 | 0.938 | 0.857 | 0.910 | 0.152 | 0.391 | 0.630 |
| Fusion_PCA | support vector machine | 0.938 (0.851-1.000) | 0.868 | 0.875 | 0.867 | 0.636 | 0.963 | 0.737 | 0.838 | 0.147 | 0.132 | 0.240 |
| Fusion_PCA | random forest | 0.967 (0.899-1.000) | 0.947 | 0.875 | 0.967 | 0.875 | 0.967 | 0.875 | 0.879 | 0.105 | 0.570 | 0.610 |
| Fusion_PCA | XGBoost | 0.858 (0.743-0.954) | 0.816 | 1.000 | 0.767 | 0.533 | 1.000 | 0.696 | 0.489 | 0.232 | 0.189 | 0.490 |
| Fusion_PLSDA | logistic regression | 0.942 (0.844-1.000) | 0.947 | 0.750 | 1.000 | 1.000 | 0.938 | 0.857 | 0.879 | 0.107 | 0.613 | 0.730 |
| Fusion_PLSDA | support vector machine | 0.942 (0.854-1.000) | 0.921 | 0.750 | 0.967 | 0.857 | 0.935 | 0.800 | 0.868 | 0.102 | 0.789 | 0.420 |
| Fusion_PLSDA | random forest | 0.967 (0.900-1.000) | 0.921 | 0.750 | 0.967 | 0.857 | 0.935 | 0.800 | 0.903 | 0.109 | 0.689 | 0.620 |
| Fusion_PLSDA | XGBoost | 0.858 (0.743-0.954) | 0.816 | 1.000 | 0.767 | 0.533 | 1.000 | 0.696 | 0.489 | 0.232 | 0.189 | 0.490 |
| Combined | Soft Voting (Clinical+CT) | 0.938 (0.848-1.000) | 0.895 | 0.875 | 0.900 | 0.700 | 0.964 | 0.778 | 0.807 | 0.118 | 0.773 | 0.380 |
