# Supplementary material for: A transfer learning-based multimodal model for early prediction of 90-day respiratory failure in dermatomyositis-associated interstitial lung disease
Source: Front Immunol. 2026 Jul 16;17:1867606. doi: 10.3389/fimmu.2026.1867606 (PMC13422525; doi:10.3389/fimmu.2026.1867606)
Supplement: Supplementary file 4 [file Table4.docx]

**Supplementary Table 4.** Stability analyses of the final early‑fusion PCA‑based RF model

| **Analysis method** | **Model** | **Mean AUC (SD)** | **Corrected AUC** |
| --- | --- | --- | --- |
| Sensitivity analysis: multiple random splits (10 splits) | Fusion_PCA RF | 0.881 (0.080) | – |
| Repeated 5‑fold cross‑validation (50 repetitions) | Fusion_PCA RF | 0.857 (0.13) | – |
| Bootstrap optimism correction (0.632+, 200 reps) | Fusion_PCA RF | – | 0.905 |

The raw test‑set AUC for the final model was 0.967 (95% CI: 0.899–1.000). Details of these stability analyses are provided in the Methods section.
