# Supplementary material for: A transfer learning-based multimodal model for early prediction of 90-day respiratory failure in dermatomyositis-associated interstitial lung disease
Source: Front Immunol. 2026 Jul 16;17:1867606. doi: 10.3389/fimmu.2026.1867606 (PMC13422525; doi:10.3389/fimmu.2026.1867606)
Supplement: Supplementary file 5 [file Table5.docx]

**Supplementary Table 5.** Performance of all candidate models in a sensitivity analysis restricted to ABG-defined cases

| **FeatureSet** | **Model** | **AUC (95% CI)** | **Accuracy** | **Sensitivity** | **Specificity** | **PPV** | **NPV** | **F1 score** | **PR-AUC** | **Brier score** | **Hosmer-Lemeshow P value** | **Optimal threshold** |
| --- | --- | --- | --- | --- | --- | --- | --- | --- | --- | --- | --- | --- |
| Clinical | LR | 0.691 (-) | 0.737 | 0.750 | 0.735 | 0.250 | 0.962 | 0.375 | 0.247 | 0.230 | 0.144 | 0.480 |
| Clinical | SVM | 0.199 (-) | 0.105 | 1.000 | 0.000 | 0.105 | 0.000 | 0.190 | 0.080 | 0.107 | 0.219 | 0.000 |
| Clinical | RF | 0.860 (-) | 0.921 | 0.500 | 0.971 | 0.667 | 0.943 | 0.571 | 0.558 | 0.114 | 0.756 | 0.700 |
| Clinical | XGB | 0.868 (-) | 0.789 | 1.000 | 0.765 | 0.333 | 1.000 | 0.500 | 0.310 | 0.231 | 0.110 | 0.470 |
| CT_PCA | LR | 0.426 (-) | 0.921 | 0.250 | 1.000 | 1.000 | 0.919 | 0.400 | 0.327 | 0.268 | 0.066 | 0.550 |
| CT_PCA | SVM | 0.294 (-) | 0.105 | 1.000 | 0.000 | 0.105 | 0.000 | 0.190 | 0.093 | 0.097 | 0.018 | 0.000 |
| CT_PCA | RF | 0.618 (-) | 0.921 | 0.500 | 0.971 | 0.667 | 0.943 | 0.571 | 0.477 | 0.201 | 0.143 | 0.580 |
| CT_PCA | XGB | 0.533 (-) | 0.421 | 0.750 | 0.382 | 0.125 | 0.929 | 0.214 | 0.110 | 0.250 | 0.152 | 0.490 |
| CT_PLSDA | LR | 0.199 (-) | 0.158 | 1.000 | 0.059 | 0.111 | 1.000 | 0.200 | 0.081 | 0.282 | 0.045 | 0.350 |
| CT_PLSDA | SVM | 0.338 (-) | 0.105 | 1.000 | 0.000 | 0.105 | 0.000 | 0.190 | 0.132 | 0.099 | 0.001 | 0.000 |
| CT_PLSDA | RF | 0.559 (-) | 0.789 | 0.500 | 0.824 | 0.250 | 0.933 | 0.333 | 0.367 | 0.262 | 0.164 | 0.680 |
| CT_PLSDA | XGB | 0.456 (-) | 0.105 | 1.000 | 0.000 | 0.105 | 0.000 | 0.190 | 0.111 | 0.252 | 0.138 | 0.000 |
| Fusion_PCA | LR | 0.551 (-) | 0.789 | 0.500 | 0.824 | 0.250 | 0.933 | 0.333 | 0.205 | 0.176 | 0.000 | 0.350 |
| Fusion_PCA | SVM | 0.301 (-) | 0.789 | 0.250 | 0.853 | 0.167 | 0.906 | 0.200 | 0.128 | 0.097 | 0.584 | 0.120 |
| **Fusion_PCA** | **RF** | **0.853 (-)** | **0.763** | **1.000** | **0.735** | **0.308** | **1.000** | **0.471** | **0.514** | **0.105** | **0.762** | **0.250** |
| Fusion_PCA | XGB | 0.882 (-) | 0.789 | 1.000 | 0.765 | 0.333 | 1.000 | 0.500 | 0.417 | 0.231 | 0.076 | 0.470 |
| Fusion_PLSDA | LR | 0.647 (-) | 0.763 | 0.750 | 0.765 | 0.273 | 0.963 | 0.400 | 0.242 | 0.204 | 0.168 | 0.450 |
| Fusion_PLSDA | SVM | 0.713 (-) | 0.895 | 0.500 | 0.941 | 0.500 | 0.941 | 0.500 | 0.509 | 0.087 | 0.518 | 0.130 |
| Fusion_PLSDA | RF | 0.853 (-) | 0.947 | 0.500 | 1.000 | 1.000 | 0.944 | 0.667 | 0.634 | 0.112 | 0.806 | 0.730 |
| Fusion_PLSDA | XGB | 0.868 (-) | 0.789 | 1.000 | 0.765 | 0.333 | 1.000 | 0.500 | 0.310 | 0.231 | 0.110 | 0.470 |
| Combined | Soft Voting (Clinical+CT) | 0.625 (-) | 0.921 | 0.500 | 0.971 | 0.667 | 0.943 | 0.571 | 0.479 | 0.205 | 0.165 | 0.530 |

Note:Due to the small sample size, the confidence intervals for AUC could not be calculated.
